# Gastric Inhibitory Polypeptide Receptor (GIPR) Overexpression Reduces the Tumorigenic Potential of Retinoblastoma Cells

**DOI:** 10.3390/cancers16091656

**Published:** 2024-04-25

**Authors:** André Haase, Emily Alefeld, Fatma Yalinci, Dario Van Meenen, Maike Anna Busch, Nicole Dünker

**Affiliations:** Center for Translational Neuro- and Behavioral Sciences, Institute of Anatomy II, Department of Neuroanatomy, Medical Faculty, University of Duisburg-Essen, 45147 Essen, Germany; andre.haase@uk-essen.de (A.H.); emily.alefeld@stud.uni-due.de (E.A.); fatma.yalinci@stud.uni-due.de (F.Y.); dariovanmeenen@uk-essen.de (D.V.M.); nicole.duenker@uk-essen.de (N.D.)

**Keywords:** retinoblastoma, RB, gastric inhibitory polypeptide receptor, GIPR, trefoil factor family peptide, TFF1, CAM, tumorigenesis, MK0893, miR-542-5p

## Abstract

**Simple Summary:**

Retinoblastoma (RB) is a malignant childhood eye cancer. In search for new or adjuvant treatment options, the gastric inhibitory polypeptide receptor (GIPR), upregulated upon the overexpression of trefoil factor family peptide 1 (TFF1), a diagnostic and prognostic biomarker for advanced RBs, came into our focus of interest. The overexpression of GIPR, found to be co-expressed with TFF1 in RB tumors, significantly reduced RB cell viability and growth and increased apoptosis levels. Moreover, GIPR-overexpressing RB cells developed significantly smaller tumors in vivo, indicating a tumor suppressor role of GIPR in RB. Although our data revealed that GIPR is not a direct TFF1 receptor, TFF1 and GIPR seem to be involved in the same signaling cascades. GIPR expression in RB cells seems to be regulated by miR-542-5p, and p53 is involved in GIPR downstream signaling, together providing potential targets for novel retinoblastoma treatment approaches.

**Abstract:**

Retinoblastoma (RB) is the most common malignant intraocular tumor in early childhood. Gene expression profiling revealed that the gastric inhibitory polypeptide receptor (GIPR) is upregulated following trefoil factor family peptide 1 (TFF1) overexpression in RB cells. In the study presented, we found this G protein-coupled transmembrane receptor to be co-expressed with TFF1, a new diagnostic and prognostic RB biomarker for advanced subtype 2 RBs. Functional analyses in two RB cell lines revealed a significant reduction in cell viability and growth and a concomitant increase in apoptosis following stable, lentiviral GIPR overexpression, matching the effects seen after TFF1 overexpression. In chicken chorioallantoic membrane (CAM) assays, GIPR-overexpressing RB cells developed significantly smaller CAM tumors. The effect of GIPR overexpression in RB cells was reversed by the GIPR inhibitor MK0893. The administration of recombinant TFF1 did not augment GIPR overexpression effects, suggesting that GIPR does not serve as a TFF1 receptor. Investigations of potential GIPR up- and downstream mediators suggest the involvement of miR-542-5p and p53 in GIPR signaling. Our results indicate a tumor suppressor role of GIPR in RB, suggesting its pathway as a new potential target for future retinoblastoma therapy.

## 1. Introduction

Affecting approximately 1 in every 18,000 live births worldwide, retinoblastoma (RB) is a rare tumor, yet it is the most common intraocular pediatric malignancy found in children under five years of age [1,2,3]. In bilateral RBs, which make up approx. 40% of all cases, the tumor effects both eyes [4]. The *RB1* gene regulates the cell cycle and inhibits tumorigenesis, and RB develops after a loss or mutation of both gene copies [1,2,4,5,6]. If diagnosed early, RB is curable, but, if left untreated, it leads to severe visual impairment and can even become life-threatening due to metastatic spread [1,7,8,9,10,11]. Enucleation, the complete removal of the affected eye, is avoided whenever salvage is possible, e.g., via intraarterial chemotherapy (IAC) or intravitreal chemotherapy (IVC). IVC focusses on precise drug delivery to the hotspot of tumor seeding, the vitreous body, reducing the toxicity of systemic chemotherapy [1,11,12,13,14,15]. However, current therapies are associated with a considerable, sometimes even complete loss of vision and a significantly increased risk for secondary tumors [16] as well as the development of chemotherapy resistance [17]. Therefore, current therapeutic and diagnostic procedures need to be improved, and new or complementary treatment methods are required.

In humans, the trefoil factor family (TFF) comprises three peptides—TFF1, TFF2, and TFF3—all possessing a characteristic clover leaf-like disulfide structure, the so-called TFF domain (for review see [18,19,20]). TFF peptides have been shown to be aberrantly expressed in a wide range of human cancer entities, including retinoblastoma (for review see [21]). Our group demonstrated that TFF1 acts as a tumor suppressor in the progression of retinoblastoma by reducing RB cell viability, growth, and proliferation and increasing apoptosis in vitro as well as inhibiting tumor growth in vivo [22]. Moreover, we previously discovered that TFF1 levels correlate with a higher clinical RB tumor-node-metastasis (TNM) stage [23], and, recently, TFF1 was described as a biomarker in retinoblastoma patients with a more advanced subtype and poor prognosis [7,24]. Most recently, we demonstrated a promising role of TFF1 as a prognostic and diagnostic marker available in the aqueous humor of RB liquid biopsies in general and under therapy in particular [25,26]. After *TFF1* overexpression in RB cells, we identified several differentially expressed genes and pathways involved in cancer progression by a gene expression array analysis [22]. One of the genes with the highest fold change in its expression levels after TFF1 overexpression was the glucose-dependent insulinotropic polypeptide or gastric inhibitory polypeptide receptor (GIPR). The human *GIPR* gene encoding for a G protein-coupled class B transmembrane protein was first cloned and molecularly characterized in 1995 [27,28,29]. G protein-coupled receptors (GPCRs) have been described as low-affinity receptors for TFF2 and TFF3 [30,31] and might be potential mediators in TFF signaling. GIPR mediates the metabolic function of the glucose-dependent insulinotropic or gastric inhibitory polypeptide (GIP), namely, glucose-dependent stimulation of insulin release from the beta cells of the pancreas ([32]; for review see [33,34]). GIPR is, however, not only expressed in the beta cells of the pancreas, throughout the gastrointestinal tract, and in adipose tissues but also in certain regions of the rat and human brain [35,36,37,38]. In the nervous system, GIP effects on neurogenesis and neuronal survival have been reported (for overview see [37]. Recently, alterations in GIPR expression have been reported in neuroendocrine tumors [39], neuroendocrine neoplasms [40], and medullary thyroid cancer [41], suggesting a clinically significant diagnostic and prognostic potential (for review see [33,34]).

As various studies reported that signaling along the GIP/GIPR axis exerts pro-proliferative and anti-apoptotic effects [42,43,44,45,46,47], in the study presented, we investigated if the effects seen after TFF1 overexpression relating to RB cell viability, cell growth, proliferation, apoptosis, and tumorigenicity are potentially mediated via the GIPR signaling axis and if GIPR might even be a TFF1 receptor yet to be found. For this purpose, we overexpressed GIPR in the RB cell lines WERI-Rb1 and Y79 and examined the effects on RB cell behavior in vitro as well as in vivo, in ovo chorioallantoic membrane (CAM) assays. Furthermore, we investigated the expression of GIPR in primary RB tumor cells in correlation with TFF1 expression and examined up- and downstream signaling components of GIPR via luciferase binding studies and a proteome profiler oncology array.

## 2. Materials and Methods

### 2.1. Human Retina and Retinoblastoma Samples

In this study, we used postmortem healthy human retinal tissue and samples of retinoblastoma patients. This research was conducted following the principles outlined in the Declaration of Helsinki. Approval for the use of human retinal tissue (approval no. 06-30214) and RB samples (approval no. 14-5836-BO) was granted by the Ethics Committee of the Medical Faculty at the University Hospital Essen, University of Duisburg-Essen. Informed consent was obtained from all the subjects involved in this study.

### 2.2. Human Cell Lines and Culture

The RB cell lines WERI-Rb1 (Weri [48] and Y79 [49], initially acquired from the Leibniz Institute DSMZ (German Collection of Microorganisms and Cell Cultures), were generously supplied by Dr. H. Stephan. The RB cell lines were cultivated as suspension cultures as described previously [50]. Human embryonic kidney (HEK293T) cells were cultivated as an adherent cell culture in DMEM medium (PAN-Biotech, Aidenbach, Germany), supplemented with 10% FBS (PAN-Biotech, Aidenbach, Germany), 4 mM L-glutamine (Gibco, Karlsruhe, Germany), 100 U penicillin/mL, and 100 μg streptomycin/mL (Gibco, Karlsruhe, Germany). The cells were maintained at 37 °C, 5% CO_2_, and 95% humidity.

### 2.3. Expression Vectors

For the construction of the GIPR overexpression vector (GIPR_plenti), the human GIPR cDNA sequence was excised from the human hGip-R pcDNA3 plasmid (cat. #14942; Addgene, Watertown, MA, USA, [28]) using the NotI fast digest restriction enzyme (Thermo Scientific, Oberhausen, Germany). Subsequently, it was ligated to the NotI-digested pENTR4 vector (cat. #17424; Addgene, Watertown, MA, USA, [51]). Afterwards, the GIPR sequence was inserted into the plenti CMV Puro Dest vector (cat. #17452; Addgene, Watertown, MA, USA [51]) using the Gateway LR Clonase II Enzyme Mix (Invitrogen, Darmstadt, Germany), following the manufacturer’s protocol. In all GIPR overexpression experiments, an empty plenti vector (empty_plenti) served as the control vector.

MicroRNA-542-5p sequences were extracted from genomic HEK293T DNA via PCR using specific primers (forward: 5′-GAATTCATTTGGGATCGGTCAAGGATG-3′; and reverse: 5′-GGATCCTTTGCTTAGGGCCCACTTTC-3′) containing EcoRI or BamHI restriction sites (underlined). After EcoRI/BamHI digestion (Thermo Scientific, Oberhausen, Germany), the miR-542-5p PCR product was integrated into the pSG5 vector (cat. #216201; Stratagene, La Jolla, CA, USA) to generate a pSG5-miR-542-5p vector. The empty pSG5 vector (pSG5) served as a control vector.

For the miR-542-5p binding studies, the wildtype miR-542-5p binding site (GIPR-BS) within the 3′-UTR of the GIPR sequence was amplified by PCR from the GIPR_plenti plasmid DNA using specific primers (forward: 5′-ACTAGTCCACACACGCTATGGAATG-3′; and reverse: 5′-GAGCTCGGGCCTTTGCCTATGCTATC-3′), containing SpeI or SacI restriction sites (underlined). Subsequently, the PCR fragments were inserted into a pCR^®^4-TOPO vector with the TOPO™TA Cloning™ Kit (Thermo Scientific; Oberhausen, Germany), following the manufacturer’s protocol. To create a mutant binding site (GIPR-MUT), primers (forward: 5′-CACTTAAGCCAGTCGACAAAGAGGTGAAAG-3′; and reverse: 5′-CTTTCACCTCTTTGTCGACTGGCTTAAGTG-3′) containing a SalI restriction site (underlined) instead of the miR-542-5p binding site were used in combination with the wildtype primers mentioned above. Following SpeI/SacI digestion (Thermo Scientific; Oberhausen, Germany), the wildtype and mutant miR-542-5p binding site PCR products were ligated into the pmiR-TK-RNL vector [52] to generate pmiR-GIPR-BS and pmir-GIPR-MUT vectors. The empty pmiR-TK-RNL vector (pmiR) served as a control vector. Validation of all the constructed vectors was performed via Sanger sequencing (Microsynth, Balgach, Switzerland).

### 2.4. Luciferase Binding Studies

The interaction between miR-542-5p and the potential binding site in the 3′-UTR of the *GIPR* gene was investigated using a Dual-Luciferase^®^ Reporter Assay System (Promega, Mannheim, Germany). HEK293T cells were transiently co-transfected with either the miR-542-5p expression vector (pSG5-miR-542-5p) or an empty control vector (pSG5), in combination with the empty pmiR-TK-RNL vector (pmiR), a vector containing the miR-542-5p binding site (pmiR-GIPR-BS), or a vector containing the mutant binding site (pmiR-GIPR-MUT). After incubation for 48 h, the cells were lysed in 1× passive lysis buffer (Promega), and luciferase activity was quantified via a dual-luciferase reporter assay (cat. #E1910, Promega, Walldorf, Germany) and visualized using a GloMax 20/20 luminometer (Promega, Walldorf, Germany) following the manufacturer’s instructions. In the assay performed, binding to the potential binding site decreased luciferase activity. The relative luciferase activity was calculated as the ratio of firefly luciferase to renilla luciferase activity. All the analyses were conducted in triplicates.

### 2.5. Transient GIPR and miR-542-5p Overexpression

For transient GIPR and miR-542-5p overexpression, 5 × 10^5^ Weri or Y79 cells were seeded into six-well plates with 2 mL DMEM (PAN-Biotech, Aidenbach, Germany) supplemented with 15% FBS (PAN-Biotech, Aidenbach, Germany) and 4 mM L-glutamine (Gibco, Karlsruhe, Germany). Plasmid DNA (4 µg) of GIPR_plenti, empty_plenti, pSG5-miR-542-5p, or empty pSG5 vectors was combined with transfection reagent (FuGENE^®^ HD; Promega, Walldorf, Germany) at a ratio of 1:5 following our previously established protocol [22].

### 2.6. Lentivirus Production and Transduction

For lentivirus production, 6 × 10^6^ HEK293T cells were co-transfected with 6 μg of each of the following plasmid DNAs: packaging vectors pczVSV-G [53], pCD NL-BH [54], and GIPR_plenti or empty_plenti, the latter serving as a negative control. Transfections were performed in the presence of 45 μg polyethyleneimine (PEI, Sigma-Aldrich, St. Louis, MI, USA) in DMEM medium. After 24 h, the medium was changed to Iscove’s Modified Dulbecco’s medium (IMDM, Pan-Biotech, Aidenbach, Germany) supplemented with 10% FBS and 1% penicillin/streptomycin. Seventy-two hours after transfection, viral supernatants were harvested, filtered (0.45 μm sterile filter), and stored at −80 °C until use.

For lentiviral transduction, 0.5 × 10^6^/mL RB cells were seeded in cell culture flasks (Greiner, Kremsmünster, Austria) in DMEM cell culture medium supplemented with 15% FBS, 4 mM L-glutamine, 100 U penicillin/mL, and 100 μg streptomycin/mL. After 24 h, the medium was replaced by GIPR (GIPR_plenti) or a negative control (empty_plenti) viral supernatant. Transduction was performed in the presence of 50 μg/mL polybrene (H9268, Sigma-Aldrich, München, Germany). After 24 h, double the volume of the DMEM medium was added to the virus supernatant. Forty-eight hours later, the medium was exchanged completely with the DMEM medium.

Virus production and transduction with TFF1 lentiviral supernatant was performed as described previously [22].

### 2.7. RNA Extraction and Quantitative Real-Time PCR

RNA was isolated using a NucleoSpin^®^ RNA II Kit (Macherey & Nagel, Düren, Germany), and microRNA was isolated using a miRNeasy Kit (Qiagen, Hilden, Germany), both following the manufacturers’ protocols.

Complementary DNA (cDNA) was synthesized using a QuantiTect Reverse Transcription Kit (Qiagen) following the manufacturer’s protocol. For quantitative Real-Time (RT) PCR analysis of *GIPR*, a SYBR™ Green PCR assay (Applied Biosystems, Dreieich, Germany) was used with specific primers (forward: 5′-GGACTATGCTGCACCCAATG-3′; and reverse: 5′-CAAAGTCCCCATTGGCCATC-3′). Human *GAPDH* (forward: 5′-ACCCACTCCTCCACCTTTGA-3′; and reverse: 5′-CTGTTGCTGTAGCCAAATTCGT-3′) served as an endogenous control. RT-PCRs were performed in triplicates using 20 µL of SYBR^TM^ Green PCR Master Mix (Applied Biosystems, Dreieich, Germany). A thermal cycling (Mastercycler X50s, Eppendorf, Hamburg, Germany) program comprised the initial denaturation step at 95 °C for 15 min, followed by 40 cycles of denaturation at 94 °C for 15 s, annealing at 55 °C for 30 s, and extension at 70 °C for 34 s.

The primers in Table 1 were used to analyze the expression levels of potential downstream targets of GIPR (see Supplementary Figure S1).

TaqMan Gene Expression Real-Time PCR analysis was performed according to the manufacturer’s protocol using the TaqMan Universal PCR Master Mix (Applied Biosystems, Dreieich, Germany). Reactions were performed in duplicate with a total volume of 20 µL and the following cycling program: 2 min at 50 °C, 10 min at 95 °C, followed by 40 cycles of 15 s at 95 °C, and 60 s at 60 °C. The following TaqMan Real-Time PCR assays (Applied Biosystems, Dreieich, Germany) were used: 18S (Hs99999901_s1), GIPR (Hs00609210_m1), and TFF1 (Hs00907239 m1).

MiRNA expression analyses were conducted using a miScript PCR Starter Kit (cat. #2181193; Qiagen, Hilden, Germany) following the manufacturer’s instructions. For miRNA quantification, miScript HiSpec buffer (Qiagen, Hilden, Germany) was used with specific primers for miR-542-5p (5′-TCGGGGATCATCATGTCACGAGA-3′) and 5.8S RNA (5′-CTACGCCTGTCTGAGCGTCGCTT-3′) as an internal control. The reactions were conducted in duplicates with the following subsequent thermal cycling program: 95 °C for 15 min, 94 °C for 15 s, 55 °C for 30 s, and 70 °C for 34 s, with a total of 40 cycles.

### 2.8. Western Blot Analyses

For the Western blot analyses, 10 × 10^6^ cells were washed with phosphate-buffered saline (PBS) and subsequently lysed in RIPA buffer supplemented according to a previously described protocol [50]. Protein extraction was achieved by ultrasonic cell lysis at 4 °C, and, afterwards, the lysates were centrifuged at 10,000× *g* at 4 °C for 30 min. The protein concentration was determined using a bicinchoninic acid assay (BCA; Thermo Scientific, Oberhausen, Germany) following the manufacturer’s instructions. Equal amounts of protein extracts were separated on a 10% SDS/PAGE gel and transferred onto nitrocellulose membranes. The membranes were blocked in 5% milk powder (Roth, Karlsruhe, Germany) and incubated overnight at 4 °C with primary antibodies against GIPR (1:2000; cat. #ab136266, Abcam, Cambridge, UK), TFF1 (1:1000; cat. #ab92377, abcam, Cambridge, UK), or β-actin (1:1000; cat. #4967; Cell Signaling Technology, Danvers, MA, USA). The blots were either cut or stripped by incubation in 0.2 N NaOH (Roth, Karlsruhe, Germany) for 15 min at RT, followed by re-blocking and incubation in an antibody solution. Horseradish peroxidase (HRP)-conjugated secondary antibodies (goat anti-rabbit; P0448, Agilent, Santa Clara, CA, USA) were applied at a dilution of 1:10,000 at room temperature for 1 h. The HRP signal was visualized with a Western Bright Chemiluminescence Reagent (Advansta, San Jose, CA, USA) and detected with a Celvin S reader (Biostep, Burkhardtsdorf, Germany).

### 2.9. Cell Viability Assays

For the determination of cell viability, a total of 4 × 10^4^ cells were seeded in 100 µL of DMEM medium into a 96-well plate in quintuplicates. Following 48 h incubation, 10 µL of a water-soluble tetrazolium (WST-1) solution (Sigma-Aldrich, St. Louis, MI, USA) was added to each well, and the cells were incubated at 37 °C for 2 h. The formazan product produced by viable cells was quantified using a microplate reader (Agilent BioTek, Santa Clara, CA, USA) at an absorbance of 450 nm.

### 2.10. Growth Kinetics

Growth kinetics analyses were performed using 24-well plates. A total of 3 × 10^5^ cells were seeded in triplicates, with each well containing 500 µL of DMEM medium (see above). The quantity of viable cells was determined by manual counts of trypan blue-stained cells at defined intervals (0, 24, 48, 72, 96, and 168 h).

### 2.11. BrdU and Caspase-3 Assays

Cell proliferation was examined by the addition of 5 µM of BrdU (5-Bromo-2′-deoxyuridine; Sigma-Aldrich, Steinheim, Germany) to the cells. Thereafter, the cells were seeded on Poly-D-Lysin (Sigma, St. Louis, MI, USA)-coated coverslips. After 4 h at 37° and 10% CO_2_, the cells were fixed in 4% paraformaldehyde (PFA). Subsequently, the cells were permeabilized with 0.1% triton X-100 (Sigma, St. Louis, MI, USA) in PBS for 30 min. To prevent unspecific binding, the cells were blocked in PBS containing 5% BSA (bovine serum albumin; Sigma, St. Louis, MI, USA) and 5% NGS (normal goat serum; Dako, Santa Clara, CA, USA). Next, the cells were incubated overnight at 4 °C with a rat anti-BrdU primary antibody (1:1000; cat. #ab6326; Abcam, Cambridge, UK). The next day the cells were washed (3 × 5 min with PBS) and incubated with an Alexa Fluor 594-labeled goat anti-rat secondary antibody (1:1000 in PBS; cat. #A-1007, Molecular Probes, Eugene, OR, USA). The number of proliferating cells was determined by manual counting.

Caspase-3-dependent apoptosis was investigated by seeding cells on coverslips and fixing them 2 h later with 4% PFA. The cells were treated with a blocking solution (5% BSA, 5% NGS, and 0.1% triton in PBS) for 1 h at room temperature, followed by incubation with a cleaved, active caspase-3 antibody (1:400; cat. #9664, Cell Signaling Technology, Danvers, MA, USA), overnight at 4 °C. The next day, the cells were washed with PBS three times (5 min each) and incubated with an Alexa Fluor 594-labeled goat anti-rabbit secondary antibody (1:1000 in PBS; cat. #A-11012, Molecular Probes, Eugene, OR, USA). The number of caspase-3-dependent apoptotic cells was determined by manual counting.

### 2.12. GIPR Inhibitor Studies

For the GIPR inhibitor studies, 4 × 10^4^ cells transduced with GIPR or control lentiviral supernatant were seeded in 100 µL of DMEM medium in a 96-well plate (Greiner, Kremsmünster, Austria). The cells were treated with the GIPR inhibitor MK0893 (MedChemExpress, Monmouth Junction, NJ, USA) diluted in DMSO (Sigma-Aldrich, Steinheim, Germany) at a final concentration of 5 nM or a DMSO (Sigma-Aldrich, Steinheim, Germany) control. Two hours later, recombinant TFF1 (rTFF1; Preprotech, Cranbury, NJ, USA) reconstituted in water was added at a final concentration of 5 µM. The controls were treated with water.

### 2.13. In Ovo Chorioallantoic Membrane (CAM) Assays

**Figure 1:**
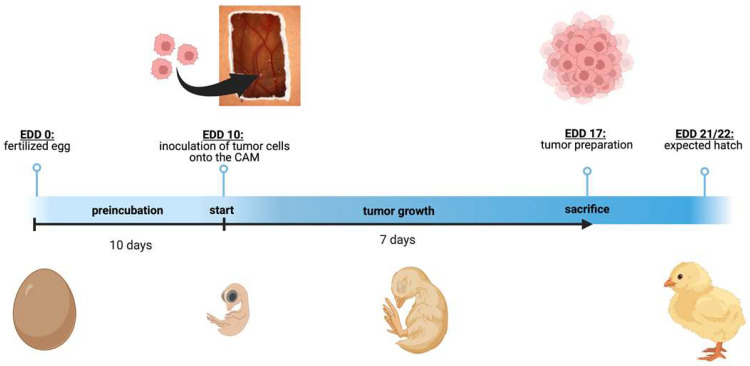
Schematic depiction of the chorioallantoic membrane (CAM) assay. Timeline of the in ovo chorioallantoic membrane (CAM) tumor cell graft model. Fertilized chicken eggs were incubated for 10 days. On embryonic development day (EDD) 10, the eggs were opened, and tumor cells were inoculated onto the CAM. On EDD 17, four days before hatching, the tumors were harvested and analyzed. The figure was created with BioRender^©^ at https://www.biorender.com (accessed on 31 October 2023).

In order to quantify changes in tumor formation capacity, tumor size, and weight of GIPR-overexpressing RB cells, 1 × 10^6^ cells transduced with GIPR or control virus particles were grafted onto the chorioallantoic membrane (CAM) of fertilized chicken eggs (see Figure 1) as described previously [50] based on the protocol of Zijlstra and Palmer [55,56]. Twenty-five fertilized eggs were grafted in at least three independent experiments. On chick embryonic developmental day (EDD) 17, seven days after grafting of the RB cells at EDD10, the tumors were excised, measured, and photographed as described previously [22,57,58].

### 2.14. Cancer-Related Protein Expression Profiling

The expression levels of 84 human cancer-related proteins were evaluated in Weri cells transduced with either GIPR or control lentiviral supernatant using the Proteome Profiler Human XL Oncology Array (R&D Systems, Minneapolis, MN, USA). The expression levels were determined in duplicate, using 200 µg of protein following the manufacturer’s protocol.

### 2.15. Statistical Analysis

The statistics were calculated using GraphPad Prism 9. The data presented represent the means ± standard error of the mean (SEM) from at least three experiments. The data were analyzed by Student’s t-test, and statistical significance was assigned for *p*-values less than 0.05 (*), 0.01 (**), 0.001 (***), or 0.0001 (****).

Statistical analyses of growth curves were performed using a web interface (http://bioinf.wehi.edu.au/software/compareCurves/, accessed on 4 December 2023). This interface allows one to compare growth curves from the statmod statistical modeling package, which is available through the “R Project for Statistical Computing” (http://www.r-project.org, accessed on 4 December 2023).

For biological pathway and gene ontology (GO) term analyses on the target genes, we used a Kyoto encyclopedia of genes and genomes (KEGG) pathway enrichment analysis, using the database for annotation, visualization, and integration discovery (DAVID) software [59]. The analysis, based on hypergeometric distribution, utilized a significance threshold of *p* < 0.05 for the selection of GO terms and pathways.

## 3. Results

### 3.1. GIPR and TFF1 Are Co-Expressed in Retinoblastoma Tumors

**Figure 2:**
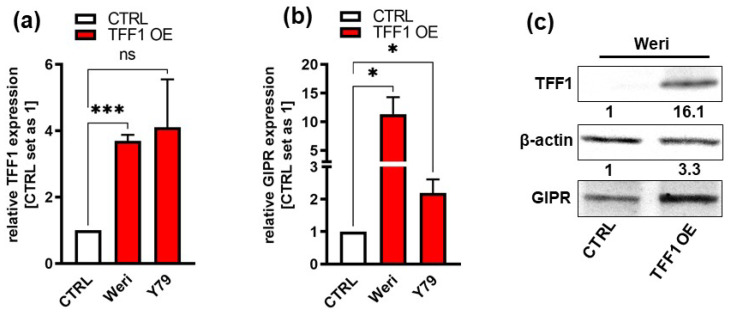
Verification of GIPR upregulation after lentiviral TFF1 overexpression in two retinoblastoma cell lines. (**a**) Verification of TFF1 overexpression in Weri and Y79 RB cells via Real-Time PCR. (**b**) After lentiviral TFF1 overexpression in two RB cell lines, GIPR is significantly upregulated at the mRNA level, as revealed by Real-Time PCR. (**c**) In TFF1-overexpressing Weri RB cells, GIPR is likewise upregulated at the protein level, as revealed by a Western blot analysis. CTRL = cells transduced with control vector; TFF1 OE = TFF1 overexpression. Values represent the means ± SEM; significances are calculated by an unpaired Student’s *t*-test. ns: not significant; * *p* < 0.05; and *** *p* < 0.001.

Previous investigations by our group revealed that the G protein-coupled receptor (GPCR) GIPR is one of the genes with the highest fold change in expression after TFF1 overexpression in RB cells. First, we verified GIPR upregulation upon successful lentiviral TFF1 overexpression (Figure 2a,c) in the RB cell lines Weri and Y79 in terms of the RNA level by Real-Time PCR (Figure 2b). In terms of the protein level, GIPR expression was, however, only significantly upregulated in Weri RB cells, as revealed by a Western blot analysis (Figure 2c). The uncropped blots and molecular weight markers are shown in Supplementary Figure S2.

**Figure 3:**
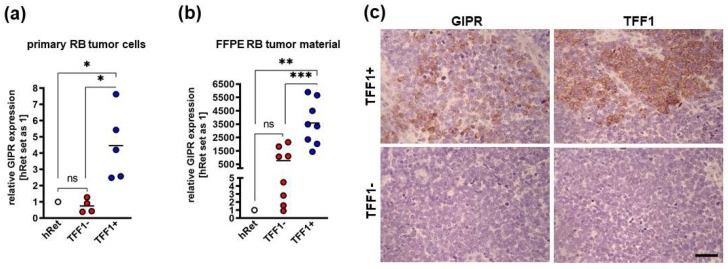
GIPR expression in TFF1-negative and TFF1-positive primary RB patient tumors. (**a**) Cultured TFF1-negative (TFF1-) patient-derived retinoblastoma (RB) tumor cells display similar GIPR mRNA levels to healthy human retina (hRet) as revealed by Real-Time PCR. By contrast, TFF1-positive (TFF1+) RB tumor cells show significantly increased GIPR expression. GIPR expression in TFF1+ primary RB tumor cells is significantly increased compared to TFF1- tumor cells. (**b**) Real-Time PCR analyses with RNA extracted from formalin-fixed paraffin-embedded (FFPE) RB patient tumors revealed that TFF1-RB tumors show higher, yet not significantly increased, GIPR mRNA levels compared to hRet, whereas TFF1+ tumors display significantly increased GIPR expression compared to hRet and TFF1-RB tumors. (**c**) Exemplary immunohistochemical stains against GIPR and TFF1 (brown) in TFF1+ and TFF1- in hematoxylin counterstained (blue) paraffin sections of RB patient tumors. Scale bar: 50 µm; applies to all pictures in C. Values represent the means ± SEM; significances are calculated by an unpaired Student’s *t*-test. ns = *p* > 0.05; * *p* < 0.05; ** *p* < 0.01; and *** *p* < 0.001.

Next, we investigated GIPR’s expression levels in retinoblastoma primary tumor tissue and correlated them with TFF1 expression. Real-Time PCR analyses revealed that cultured primary RB patient-derived tumor cells, which do not express TFF1 (TFF1-negative; TFF1) displayed similar GIPR levels to healthy human retina (hRet; Figure 3a). TFF1-expressing (TFF1-positive; TFF1+) RB tumor cells, by contrast, showed significantly increased GIPR expression compared to TFF1-RB tumor cells and compared to hRet (Figure 3a). Formalin-fixed paraffin-embedded TFF1-RB patient tumors displayed higher, yet not significantly increased, GIPR levels compared to hRet, whereas TFF1+ tumors showed significantly increased GIPR expression compared to hRet and TFF1-RB tumors (Figure 3b). Exemplary immunohistochemical stains of RB patient tumor sections revealed that TFF1+ tumors are also positive for GIPR, whereas TFF1-tumors also stain negatively for GIPR (Figure 3c). The co-expression of GIPR and TFF1 in RB patient tumors, which did express TFF1, allowed potential TFF1 signaling via the GIPR receptor.

### 3.2. GIPR Overexpression Results in Decreased Cell Viability, Cell Growth, and Proliferation as Well as Increased Apoptosis in RB Cell Lines In Vitro

**Figure 4:**
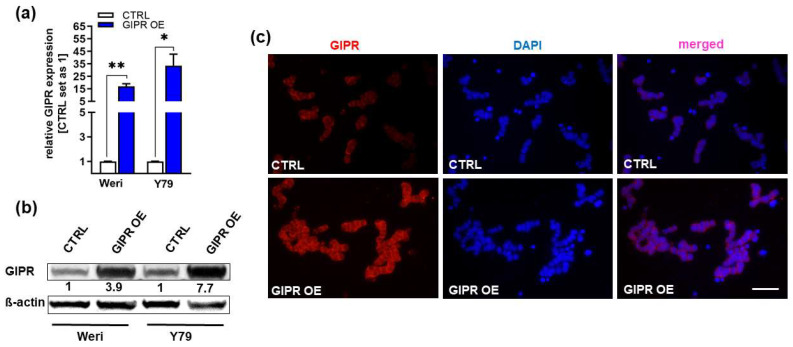
Verification of GIPR overexpression in retinoblastoma cell lines. (**a**) Verification of GIPR overexpression (OE) in the retinoblastoma (RB) cell lines Weri and Y79 on mRNA level via Real-Time PCR. (**b**) Verification of GIPR overexpression at the protein level via Western blot analysis in the RB cell lines Weri and Y79. (**c**) Immunofluorescent stains against GIPR (red fluorescence) with DAPI (blue) counterstaining after GIPR overexpression in Weri RB cells. Scale bar: 50 µm (applies to all pictures in (**c**). CTRL = cells transduced with control vector; GIPR OE = GIPR overexpression. Values represent the means ± SEM; significances are calculated by an unpaired Student’s *t*-test. * *p* < 0.05; ** *p* < 0.01.

In order to investigate if the decrease in RB cell viability, cell growth, proliferation, and tumorigenicity and the increase in apoptosis seen after TFF1 overexpression might be mediated via the GIPR signaling axis, we transduced GIPR in the RB cell lines Weri and Y79, generating stably GIPR-overexpressing cells. Successful GIPR overexpression was verified by Real-Time PCR (Figure 4a), Western blot analysis (Figure 4b), and immunofluorescence staining (Figure 4c). The uncropped blots and molecular weight markers are shown in Supplementary Figure S3.

**Figure 5:**
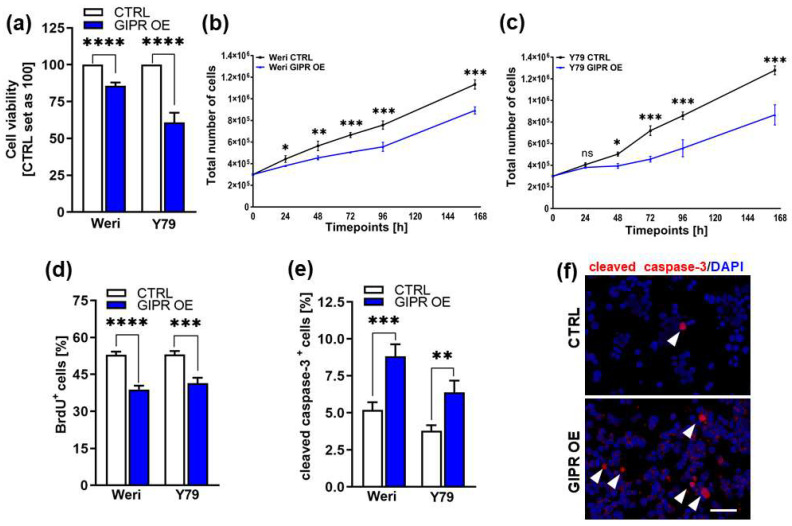
In vitro effects of GIPR overexpression in retinoblastoma cell lines. (**a**) Cell viability was significantly decreased following GIPR overexpression (GIPR OE; blue bars) in the retinoblastoma (RB) cell lines Weri and Y79, as revealed by WST-1 assays. (**b**,**c**) Growth kinetics of Weri (**b**) and Y79 (**c**) RB cells were significantly decreased after GIPR overexpression. (**d**) Proliferation of Weri and Y79 cells was significantly decreased after GIPR overexpression, as revealed by the quantification of BrdU stains. (**e**) The significant increase in apoptosis after GIPR overexpression in Weri and Y79 cells was caspase-3-dependent, as revealed by the quantification of immunocytochemical stains against cleaved caspase-3. (**f**) Immunocytochemical stains against cleaved caspase-3 in Weri control cells (CTRL) and GIPR-overexpressing (GIPR OE) Weri cells. Arrowheads indicate cleaved caspase-3-positive (cleaved caspase-3^+^) cells. Scale bar 50 µm. CTRL = cells transduced with control vector. Values represent the means ± SEM; significances were calculated by an unpaired Student’s *t*-test. ns = *p* > 0.05; * *p* < 0.05; ** *p* < 0.01; *** *p* < 0.001; and **** *p* < 0.0001.

Cell viability was significantly decreased after GIPR overexpression in both of the RB cell lines investigated as revealed by WST-1 viability assays (Figure 5a). Accordingly, our growth curve analyses showed significantly diminished growth rates of Weri and Y79 GIPR-overexpressing cells compared to the control cells (Figure 5b,c) and the proliferation levels of both cell lines were also decreased, as revealed by the BrdU cell counts (Figure 5d,e). Additionally, a significant increase in caspase-3-dependent apoptosis was seen upon GIPR overexpression in both cell lines, as revealed by the quantification of immunofluorescent staining against cleaved caspase-3 (Figure 5f). In summary, the impact of GIPR overexpression mirrors the effects previously seen upon TFF1 overexpression, indicating potential TFF1 signaling via the GIPR axis.

### 3.3. GIPR-Overexpressing RB Cells Form Significantly Smaller Tumors In Vivo

**Figure 6:**
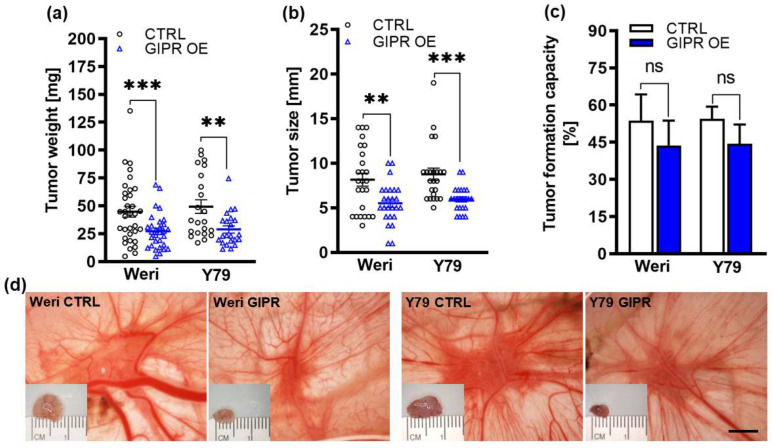
In vivo effects of GIPR overexpression in in ovo CAM assays. (**a**–**c**) Quantification of weight, size, and formation capacity of CAM tumors developing from GIPR-overexpressing (GIPR OE) Weri and Y79 RB cells inoculated in the CAM. (**d**) Representative pictures of RB CAM tumors in situ and excised tumors on a ruler (insets). GIPR-overexpressing RB cells form significantly smaller tumors in the in ovo CAM assay. Scale bar in (**d**): 5 mm; applies to all pictures. CTRL = cells transduced with control vector. Values represent the means ± SEM; significances are calculated by an unpaired Student’s *t*-test. ns = *p* > 0.05; ** *p* < 0.01; and *** *p* < 0.001.

Next, we used the chicken chorioallantoic membrane (CAM) assay to examine the impact of GIPR overexpression on RB cell tumor growth and formation capacity in an in vivo model (for the schematic depiction, see Figure 1). Stably GIPR-overexpressing Weri and Y79 cells were inoculated into the CAM of 10-day-old chicken embryos. The quantification of CAM tumor weight and size revealed that both GIPR-overexpressing RB cell lines investigated formed significantly lighter and smaller tumors in ovo than the control cells (Figure 6a,b,d). Compared to the controls, the tumor formation capacity was not significantly changed in the GIPR-overexpressing Weri and Y79 cells (Figure 6c).

### 3.4. Impact of the Administration of a GIPR Inhibitor and/or Recombinant TFF1 on Cell Viability, Proliferation, and Cell Death of GIPR-Overexpressing RB Cell Lines

Next, we set out to address the question of whether the effects seen after GIPR overexpression are specific and if GIPR might be a receptor for TFF1. For this purpose, we blocked GIPR signaling after its overexpression in two RB cell lines with a specific inhibitor (MK0893) and treated the cells with recombinant TFF1 (rTFF1) alone or in combination with MK0893 and analyzed effects on cell viability, proliferation, and apoptosis.

**Figure 7:**
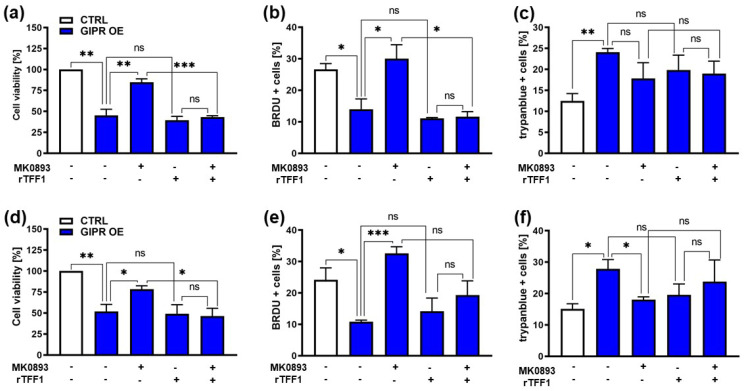
Effects of administration of a GIPR inhibitor and recombinant TFF1 on GIPR-overexpressing RB cells. (**a**,**d**) Cell viability was significantly decreased following GIPR overexpression (GIPR OE; blue bars) in the retinoblastoma (RB) cell lines Weri (**a**) and Y79 (**d**), as revealed by WST-1 assays after 24 h. After the administration of the GIPR inhibitor MK0893, the effect was reversed. The addition (+) of recombinant TFF1 (rTFF1) or a combination of MK0893 and rTFF1 did not lead to changes in cell viability compared to untreated GIPR-overexpressing cells (−). (**b**,**e**) Cell proliferation in Weri (**b**) as well as in Y79 (**c**) cells was decreased after GIPR overexpression, as revealed by the quantification of BrdU stains. Following the administration of MK0893, the effect was reversed, and the proliferation levels exceeded those of the control cells, transduced with a control vector (CTRL). The addition of rTFF1 did not lead to changes in proliferation compared to the untreated GIPR-overexpressing cells. (**c**,**f**) Changes in the cell death levels after GIPR overexpression were revealed by the counting of trypan blue-positive cells. GIPR overexpression resulted in an increased apoptosis level of Weri (**c**) and Y79 (**f**) cells. Following the administration of MK0893, the cell death levels dropped significantly in Y79 (**f**) but not in the Weri cell line. The addition of rTFF1 did not lead to significant changes compared to the cell death levels of the untreated controls. The legends in a and d also apply to all the other graphs. Values represent the means ± SEM; significances are calculated by an unpaired Student’s *t*-test. ns = *p* > 0.05; * *p* < 0.05; ** *p* < 0.01; and *** *p* < 0.001.

The significant reduction in cell viability and proliferation as well as the induction of apoptosis seen after GIPR overexpression in the retinoblastoma cell lines Weri and Y79 were significantly reversed upon the administration of MK0893 (Figure 7a–f), indicating that the effects seen after GIPR overexpression on RB cells were specific. The treatment of GIPR-overexpressing RB cells with rTFF1 did not change the cell viability, proliferation, or apoptosis levels compared to those of untreated GIPR-overexpressing cells (Figure 7a–f). Thus, no additive or synergistic effect due to the binding of TFF1 to the upregulated GIPR receptor levels in GIPR-overexpressing cells could be observed. By contrast, the combined treatment with the GIPR inhibitor and rTFF1 resulted in a significant decrease in cell viability and proliferation compared to the administration of the GIPR inhibitor alone (Figure 7a–e), indicating that the effects on cell viability and proliferation induced by TFF1 were independent of GIPR. However, an induction of apoptosis upon treatment with rTFF1 in comparison to an induction of apoptosis by MK0893 alone could not been detected (Figure 7c,e). Although our data revealed that GIPR is not a direct TFF1 receptor, the fact that GIPR is upregulated after TFF1 overexpression and that the same effects are induced upon GIPR and TFF1 overexpression indicate that TFF1 and GIPR are involved in the same signaling cascades.

### 3.5. GIPR Expression in Retinoblastoma Cells and Its Regulation by miR-542-5p

**Figure 8:**
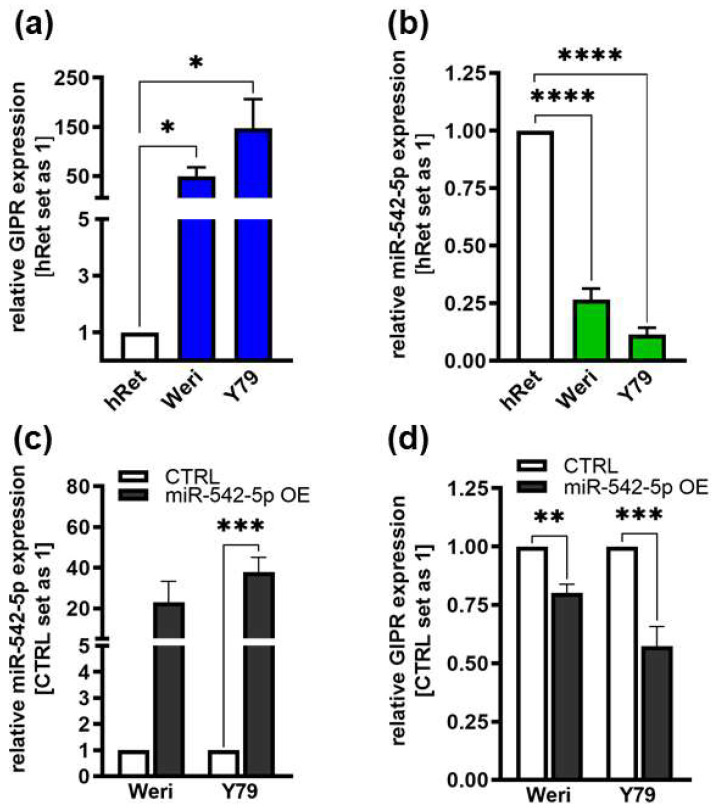
Endogenous GIPR and miR-542-5p expression levels in RB cell lines and expression after miR-542-5p overexpression. (**a**,**b**) Compared to healthy human retina (hRet), the RB cell lines Weri and Y79 displayed increased GIPR ((**a**); blue bars) and decreased miR-542-5p ((**b**); green bars) mRNA expression levels, as revealed by Real-Time PCR. (**c**,**d**) After successful miR-542-5p overexpression (grey bars; (**c**)), verified by Real-Time PCR, the RB cell lines displayed significantly decreased GIPR mRNA expression levels (**d**). CTRL = cells transduced with control vector. miR-542-5p OE = miR-542-5p overexpression. Values represent the means ± SEM; non-significant *p*-values are not shown. * *p* < 0.05; ** *p* < 0.01; *** *p* < 0.001; and **** *p* < 0.0001.

As GIPR has previously been described as a potential target gene of miR-542-5p [60], we analyzed the expression of this miR and GIPR in Weri and Y79 RB cells in comparison to healthy human retina. Compared to healthy human retina, GIPR expression was significantly increased in both of the RB cell lines investigated (Figure 8a), whereas the miR-542-5p expression levels were significantly decreased (Figure 8b). The opposing expression of miR-542-5p and GIPR suggests that the GIPR levels in RB cells might be regulated by mir-542-5p. This hypothesis was supported by the observation that GIPR expression significantly decreased (Figure 8d) upon transient mir-542-5p overexpression (Figure 8c) in Weri and Y79 cells. In order to analyze if TFF1 is also involved in the miR-542-5p GIPR signaling axis, we additionally investigated the expression of TFF1 after miR-542-5p overexpression as well as the miR-542-5p expression levels after TFF1 overexpression. Our data, however, did not reveal a regulatory mechanism between miR-542-5p and TTF1 (Supplementary Figure S4).

**Figure 9:**
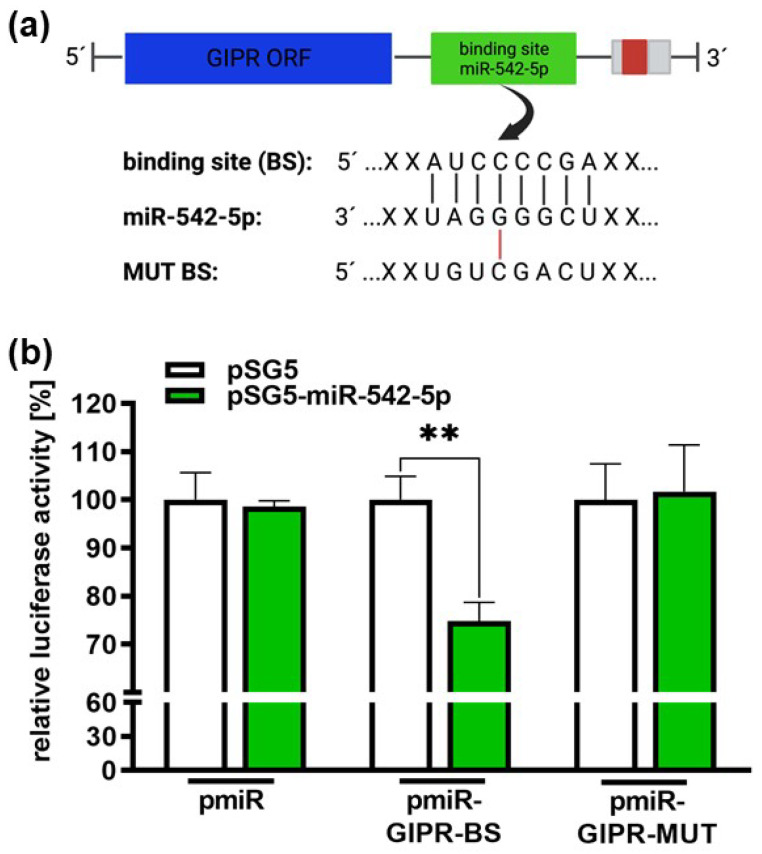
Binding studies of miR-542-5p to the 3′-UTR region of GIPR. (**a**) Depiction of potential binding sites of miR-542-5p at the 3′-UTR region of the GIPR gene. The GIPR 3′-UTR region contains a potential binding site for miR-542-5p adjacent to the open reading frame (GIPR ORF). MiR-542-5p can bind to the potential binding site (BS), whereas it cannot bind to the mutant binding site (MUT BS). (**b**) For the luciferase binding studies, HEK293T cells were co-transfected with a miR-542-5p expression vector (pSG5-miR542-5p) in addition to either a wildtype (pmiR-GIPR-BS) or mutant (pmiR-GIPR-MUT) vector containing the binding sequence of the GIPR 3′-UTR. Empty vectors (pSG5 and pmiR) served as the controls. After 48 h, decreased luciferase activity indicated the binding of miR-542-5p to the 3‘-UTR of the GIPR gene (pmiR-GIPR-BS). No binding was observed for the mutant GIPR binding site (pmiR-GIPR-MUT). The values are the means of at least three independent experiments ± SEM; significances are calculated by an unpaired Student’s *t*-test. Non-significant *p*-value calculations are not shown. ** *p* < 0.01. The figure was created with BioRender^©^ at https://www.biorender.com (accessed on 31 October 2023).

Target scans predicted a potential binding site for miR-542-5p within the 3′-UTR region of the *GIPR* gene (Figure 9a). Luciferase activity assays were performed to assess the binding of miR-542-5p to the GIPR 3′-UTR region. Our binding study revealed that miR-542-5p binds to the wildtype binding site of GIPR in the 3′-UTR detected by significantly reduced luciferase activity (Figure 9b). As a control for the binding specificity, we mutated the GIPR binding site (Figure 9a) and measured the binding of miR-542-5p. In this setting, luciferase activity remained unchanged compared to the binding of the empty vector control (Figure 9b), indicating that miR-542-5p has the capability to specifically bind to and regulate GIPR expression in RB cell lines.

### 3.6. GIPR Downstream Signaling Targets

**Figure 10:**
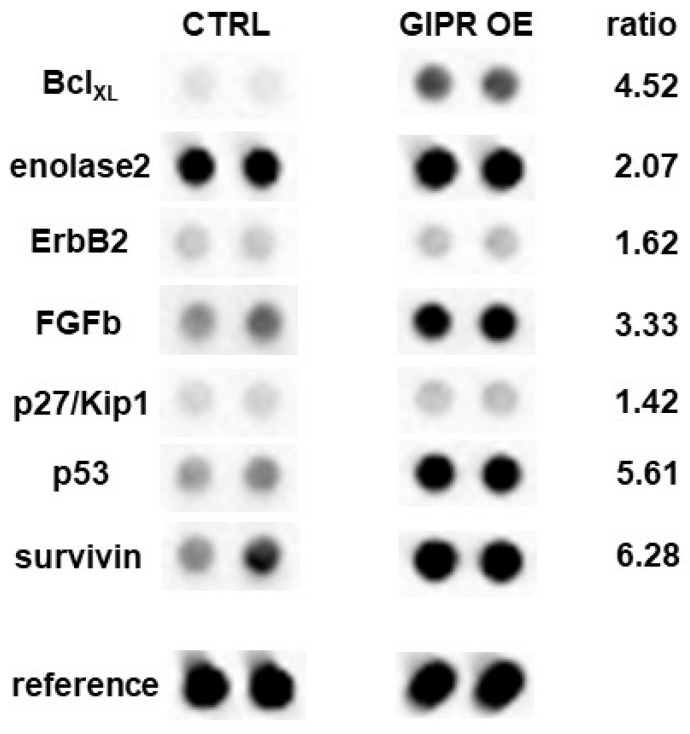
Expression of tumor-related proteins after GIPR overexpression in Weri cells as revealed by a human oncology array. Duplicate spots of differentially expressed proteins are shown. Grey-scale intensities were recorded and the ratios for the control cells vs. the GIPR-overexpressing cells were calculated. CTRL = cells transduced with control vector; GIPR OE = GIPR-overexpressing cells.

To gain a deeper insight into GIPR downstream signaling in RB cells, cancer-associated proteins were analyzed in a human oncology array. Following GIPR overexpression 9 out of 84 proteins were differentially regulated in Weri RB cells: BCL_XL_, enolase 2, ErbB2, FGFb, p27/Kip1, p53, and survivin were upregulated, whereas MMP3 was downregulated compared to the controls (Figure 10). The whole human oncology arrays are shown in Supplementary Figure S5.

Differential expression of these proteins was confirmed at mRNA level via Real-Time PCR (Supplementary Figure S1). In Y79 cells, no significant regulation of the before-mentioned proteins was seen at the protein level. At the mRNA level, by contrast, all the proteins upregulated in the Weri cells were likewise upregulated; however, the levels did not reach significance (Supplementary Figure S1).

DAVID analyses of all the proteins differentially expressed in the Weri cells revealed 10 significantly enriched GO terms (Table S1) and 18 KEGG pathways with at least three counts and *p* < 0.05 (Table S2). Most of the GO terms were related to “apoptosis”, “proliferation”, “cell migration”, “cell cycle”, and “angiogenesis”, all processes playing an important role in cancer and shown to be affected by GIPR overexpression in RB cells. Nine out of the eighteen identified KEGG pathways were associated with cancer.

## 4. Discussion

We previously discovered that TFF1 overexpression in RB leads to anti-tumorigenic effects, suggesting a potential tumor suppressor role of TFF1 in this tumor entity [22,23]. Upon TFF1 overexpression, we found GIPR to be one of the highest differentially regulated genes and hypothesized that TFF1′s effects on cell viability, growth, proliferation, apoptosis, and tumorigenicity in RB cells might be mediated via the GIPR signaling axis and that GIPR might as well be a receptor for TFF1. Most recently, TFF1 was also identified as an RB biomarker for a subset of more advanced RBs [7,23,24], being detectable in the aqueous humor of RB patients [25,26]. At a first glance, a potential tumor suppressor function of TFF1 and elevated expression levels in more advanced, higher-metastasizing RB tumors seem to be conflicting. However, the two facts are not necessarily contradictory, as various mechanisms potentially upregulate a tumor suppressor in advanced tumors. Possible scenarios are, e.g., cellular stress response to hypoxia and high reactive oxygen species (ROS) levels, frequently observed in more advanced tumor stages, or the activation of the immune system by inflammation, a key player in carcinogenesis. Fittingly, the ectopic expression of TFF1 during chronic inflammation processes and a role of TFF1 as an ROS scavenger have been previously described for various tissues (for review, see [18]). In addition, genetic alterations including mutations or altered epigenetic regulations are potential mechanisms explaining the discrepancy described above. Along this line, we could show that TFF1 is epigenetically regulated in RB [61], and others observed a correlation between cancer progression and mutations/polymorphisms in the TFF1 gene [62,63,64,65,66]. To gain a deeper understanding of the molecular mechanisms underlying the dual tumor suppressor and biomarker function of TFF1, it is important to gain a deeper insight into its signaling pathways.

Thus, we investigated the general function of GIPR signaling in RB and its correlation with TFF1. In the study presented, we observed significantly elevated GIPR levels in Weri and Y79 RB cells compared to healthy human retina. In a pathological context, human and rat medullary thyroid cancers display high GIPR expression levels compared to normal tissue, and massive overexpression of GIPR was described for neoplastic C cells of both rats and humans [41]. Moreover, GIPR is significantly overexpressed in various neuroendocrine tumors (NETs) compared to normal tissue [67,68]. Particularly, pancreatic, illeal, and bronchial NETs display very high GIPR expression [39]. Moreover, while TFF1-negative RB tumor cells displayed similar GIPR levels to healthy human retina, significantly increased GIPR expression levels were detected in TFF1-positive primary tumor cells, representing the subset of more advanced RBs with TFF1 as an indicating biomarker [7,24]. Additionally, we verified the upregulation of GIPR’s levels upon TFF1 overexpression in Weri and Y79 cells at the RNA and protein level, indicating a possible functional correlation of both proteins. In neuroendocrine neoplasms (NENs), high GIPR expression likewise correlates with a high tumor grade. In these tumor entities, GIPR levels gradually increase in a subset of insulinomas and non-functioning pancreatic NENs [40]. Furthermore, increased GIPR expression has been correlated with liver metastasis [69]. Further along this line, Costa et al. observed significantly higher GIPR mRNA levels in malignant adrenocortical carcinomas than in benignant adenomas in both pediatric and adult patients [70]. Moreover, the presence of GIPR was demonstrated in advanced colorectal cancer (CRC) and MC-26 and HT29 cells, two CRC cell lines [43]. Interestingly, neither epithelial and stromal gastrointestinal (GI) tumors and GI stromal tumors nor lung adenocarcinomas express GIPR, except for a subgroup of pancreatic adenocarcinomas [39]. Our data support the hypothesis that TFF1 and GIPR, both expressed in higher-grade RB tumors, may be involved in the same signaling pathways.

To further study the functional role of GIPR in RB, we overexpressed GIPR in Weri and Y79 retinoblastoma cells. Increased GIPR expression resulted in significantly reduced cell viability, cell growth, and proliferation, and significantly smaller tumors were formed in in vivo CAM assays as well as significantly increased caspase-3-dependent cell death levels in vitro, mirroring the effects previously seen after TFF1 overexpression [22] and indicating a role of this protein as a tumor suppressor. In previous studies, GIPR signaling was instead mainly linked to the survival of pancreatic ß cells [45]. Contrasting our findings, Campbell et al. demonstrated that pancreatic ß cells from *Gipr*^−/−ßCell^ mice with a selective ablation of GIPR displayed a significantly higher sensitivity to apoptosis [45]. Further along this line, in vitro studies in ß-insulin (INS) cells showed that GIP stimulation protected these cells against streptozotocin-induced apoptosis [46]. Moreover, GIP has been shown to promote ß-(INS) cell survival [47] and stimulate the proliferation of MC-26 and HT29 CRC cells expressing the GIPR [43], contradicting the findings of our study, where high GIPR levels after GIPR overexpression reduced cell viability and growth. This discrepancy might be explained in terms of comparing the effects of metabolic signaling along the GIP/GIPR axis with the pathological conditions of a cancer cell line. Otherwise, the effects might be tissue-dependent, as very high GIPR levels in neuroendocrine tumors have been observed to either increase or decrease raised proliferation levels, depending on the tumor site [39,40].

Next, we set out to investigate how GIPR expression is regulated in RB tumor cells. In non-small-cell lung cancer (NSCLC), GIPR has been described as 1 of 457 potential target genes of miR-542-5p [60]. Moreover, it has been shown that pristimerin, a natural-occurring quinone methide triterpenoid with anticancer effects, inhibits glioblastoma progression by targeting two receptors, the protein tyrosine phosphatase, non-receptor type 1 (PTPN1), and Argonaute 2 (AGO2) via miR-542-5p [71]. Target scans confirmed GIPR as a potential target of this miR, but it has not been experimentally proven so far. In the study presented in this paper, we observed an opposing expression pattern of miR-542-5p and GIPR expression, with GIPR expression being significantly higher in Weri and Y79 RB cells and miR-542-5p levels being significantly lower compared to those in healthy human retina. Upon miR-542-5p overexpression, the GIPR significantly decreased, suggesting that miR-542-5p plays a role in regulating GIPR expression in RB cells. To further address this hypothesis, we performed luciferase binding studies and were able to prove the direct binding of miR-542-5p to the 3′-UTR of the *GIPR* gene. Thus, GIPR expression in RB cells is most likely at least partially regulated by miR-542-5p, without the involvement of TFF1.

A TFF receptor remained unknown for a long time, until, in 2009, the chemokine receptor type 4 (CXCR4), which belongs to the G protein-coupled receptor family (GPCR), was described as a low-affinity receptor for TFF2 [30]. Moreover, Dieckow et al. could show that CXCR4 and CXCR7 are involved in the TFF3-dependent activation of cell migration [31]. Therefore, GPCRs like GIPR, involved in various diseases and, consequently, targets of over 40% of drugs currently on the market [72,73], are potential mediators of TFF signaling. To address the question of whether GIPR is involved in TFF1 signaling as a direct TFF1 receptor, in the study hereby presented, we performed GIPR inhibitor experiments and stimulated the cells with recombinant TFF1 (rTFF1). We could show that the effects of GIPR overexpression are specific, since the reduction in cell viability seen in GIPR-overexpressing cells was reversed upon the administration of the GIPR inhibitor. However, the reduced cell viability in GIPR-overexpressing cells induced by rTFF1 could not be reversed by inhibiting GIPR, indicating that this effect is not GIPR-dependent and, thus, that GIPR is most likely not a direct TFF1 receptor.

Subsequently, we investigated the downstream targets of GIPR signaling in RB cells via a human oncology array, revealing p53 as one of the upregulated proteins in Weri and Y79 RB cells. The tumor suppressor gene *TP53* is, for instance, involved in the regulation of cell cycle arrest and apoptosis [74,75,76]. Our group already demonstrated that TFF1 induces apoptosis and decreases proliferation and tumor growth in human retinoblastoma cell lines in a p53-dependent manner [22]. Thus, upregulated p53 levels following GIPR overexpression would support the hypothesis that TFF1′s effects are mediated via GIPR signaling. Since it has been shown that p53 induces cell death via the transcriptional activation of the pro-apoptotic protein Bax [77] or direct binding to Bcl2 and Bcl_XL_ [78], we also investigated the expression of these proteins. In our Western blot analyses, we did not observe any significant changes in the protein expression levels of Bax and Bcl2 following GIPR overexpression in Weri and Y79 cells. Bcl_XL_, on one hand, was found to be upregulated by GIPR overexpression in Weri and Y79 cells. In our setting, increased levels of the anti-apoptotic protein Bcl_xL_ at a first glance did not correlate with increased apoptosis levels following GIPR overexpression in RB cells. However, since p53 was also upregulated after GIPR overexpression, a possible scenario fitting our effects could be a direct induction of mitochondrial outer-membrane permeabilization (MOMP) via interaction with anti-apoptotic Bcl_xL_, which, in turn, would lead to caspase-dependent apoptosis [78]. This would confirm our previously shown results, according to which TFF1 induces the apoptosis of human RB cell lines in a caspase-dependent manner [22]. Fittingly, in the study hereby presented, the increase in apoptosis seen after GIPR overexpression in RB tumor cells was likewise caspase-3-dependent. In addition, it has been demonstrated that the GIP-mediated suppression of apoptosis in a pancreatic ß-insulin cell line is caspase-3-dependent [42,46]. Moreover, “regulation of apoptosis” was a significantly enriched GO term in GIPR-overexpressing Weri RB cells, and “apoptosis” was one of the enriched KEGG pathways.

In conjunction with apoptosis regulation, we found survivin, an anti-apoptotic family member of the inhibitor of apoptosis proteins (IAPs; for review, see [79]), to be upregulated in GIPR-overexpressing Weri cells. Survivin is overexpressed in various tumor entities, and its overexpression frequently correlates with cancer progression and recurrence [79]. Increased levels of survivin, however, do not correlate with increased apoptosis levels and reduced tumorigenicity following GIPR overexpression in Weri RB cells. These discrepancies might be explained in terms of the counter-regulatory mechanisms induced by the pro-apoptotic effects seen after GIPR overexpression.

In the study hereby presented, fibroblast growth factor 2 (FGF2/FGFb) was likewise upregulated upon GIPR overexpression in RB cells. FGFs are known as key factors in tissue homeostasis and cancer. FGFb regulates the self-renewal of multiple stem cell types and plays a pivotal role in brain tumors, particularly in malignant glioma [80]. Downstream signaling involves the FGF receptor family, PI3K/AKT, and RAS/RAF/MAPK, which also exert pro-proliferative and anti-apoptotic effects during metabolic signaling along the GIP/GIPR axis [42,43,44,81]. Therefore, we analyzed the phosphorylation status of Akt and the MAP kinase ERK1/2 after GIPR overexpression in Weri and Y79 RB cells; however, we observed no obvious changes in phosphorylation. In line with these findings, in medullary thyroid cancer, the cell effects of GIPR receptor stimulation on the downstream PI3K-Akt and MAPK-ERK1/2 signaling axis were likewise only marginal [34]. How upregulated FGFb expression fits into these signaling pathways and the GIPR-mediated effects seen in RB cells remains to be further investigated.

In summary, we identified GIPR as a potential key player involved in TFF1 signaling, triggering tumor-suppressing effects in RB, most likely with the involvement of miR-542-5p and p53 as up- and downstream mediators.

## 5. Conclusions

In the study hereby presented, the stable overexpression of the G protein-coupled transmembrane receptor GIPR, shown to be upregulated following the overexpression of TFF1, resulted in significantly increased apoptosis levels and a concomitant decrease in cell viability, growth, and proliferation in vitro as well as tumor growth in vivo, suggesting a tumor suppressor role of GIPR in RB. Although our data indicate that GIPR is not a receptor for TFF1, TFF1 and GIPR seem to be involved in the same signaling cascades, and up- and downstream signaling mediators like miR-542-5p and p53 are potential targets for new retinoblastoma treatment approaches. In future experiments, these novel treatment and adjuvant therapy options, e.g., modified nanoparticles, should be tested using in ovo and in vivo rodent models in order to optimize future RB treatment.

## Figures and Tables

**Table 1:** Real-Time PCR primers for GIPR downstream signaling analyses.

Primer	5′-3′ Sequence
ENO2 FW	CTACCACCGTCTGAGTCTGC
ENO2 RV	CCTTCAGGACACCTTTGCCT
ERBB2 FW	GTTCCCGGATTTTTGTGGGC
ERBB2 RV	GTGGTACTTCAATTGCGACTCA
p27 FW	CTGCAACCGACGATTCTTCT
p27 RV	GCATTTGGGGAACCGTCTGA
p53 FW	TGTGACTTGCACGTACTCCC
p53 RV	ACCATCGCTATCTGAGCAGC
Survivin FW	TGAGAACGAGCCAGACTTGG
Survivin RV	TGGTTTCCTTTGCATGGGGT
FGFb FW	CCGTTACCTGGCTATGAAGG
FGFb RV	AAAGAAACACTCATCCGTAACACA

## Data Availability

The data presented in this study are available upon request from the corresponding author.

## Supplementary Materials

The following supporting information can be downloaded at https://www.mdpi.com/article/10.3390/cancers16091656/s1, Figure S1: Verification of human oncology array data via Real-Time PCR; Figure S2: Uncropped Western blots shown in Figure 2c; Figure S3: Uncropped Western blots shown in Figure 4b; Figure S4: Hypothetical TFF1-miR-542-5p or miR-542-5p-TFF1 signaling axis; Figure S5: Uncropped human oncology array shown in Figure 10; Table S1: Significantly enriched GO terms of genes differentially expressed after GIPR overexpression in Weri RB cells; Table S2: Significantly enriched KEGG pathways of genes differentially expressed after GIPR overexpression in Weri RB cells.
