# Supplementary material for: Gastric Inhibitory Polypeptide Receptor (GIPR) Overexpression Reduces the Tumorigenic Potential of Retinoblastoma Cells
Source: Cancers (Basel). 2024 Apr 25;16(9):1656. doi: 10.3390/cancers16091656 (PMC11083251; doi:10.3390/cancers16091656)
Supplement: Supplementary file 1 [file cancers-16-01656-s001.zip › cancers-2953080-supplementary.pdf]

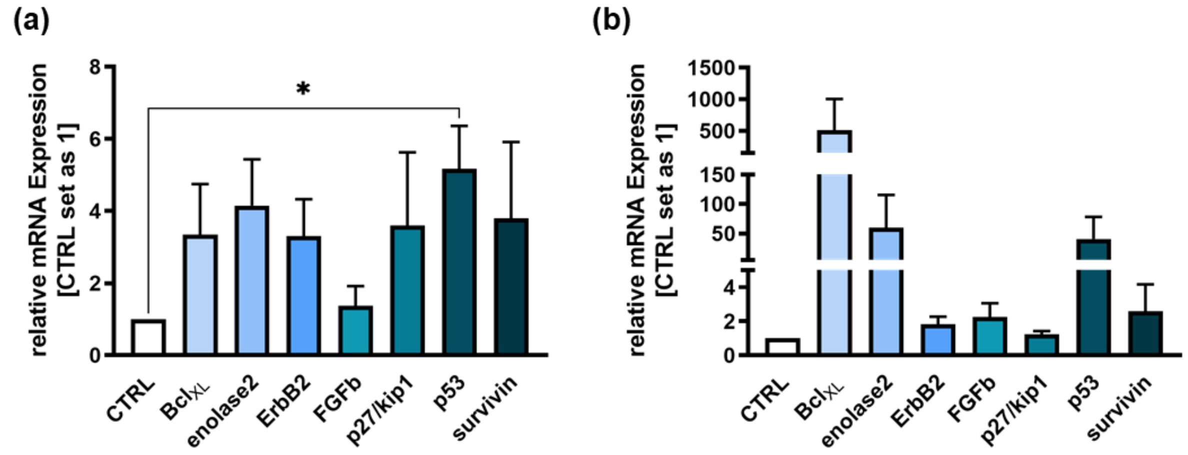


**Figure S1.** Verification of human oncology array data via Real-Time PCR. Potential GIPR pathway downstream signaling molecules found to be upregulated on protein level upon GIPR overexpression were likewise upregulated on mRNA level in WERI (**a**) and Y79 RB cells (**b**). white bar: CTRL= cells transduced with control vector. Colored bars: GIPR overexpressing cells. Values represent means ± SEM; not significant p-values are not shown. *p<0.05

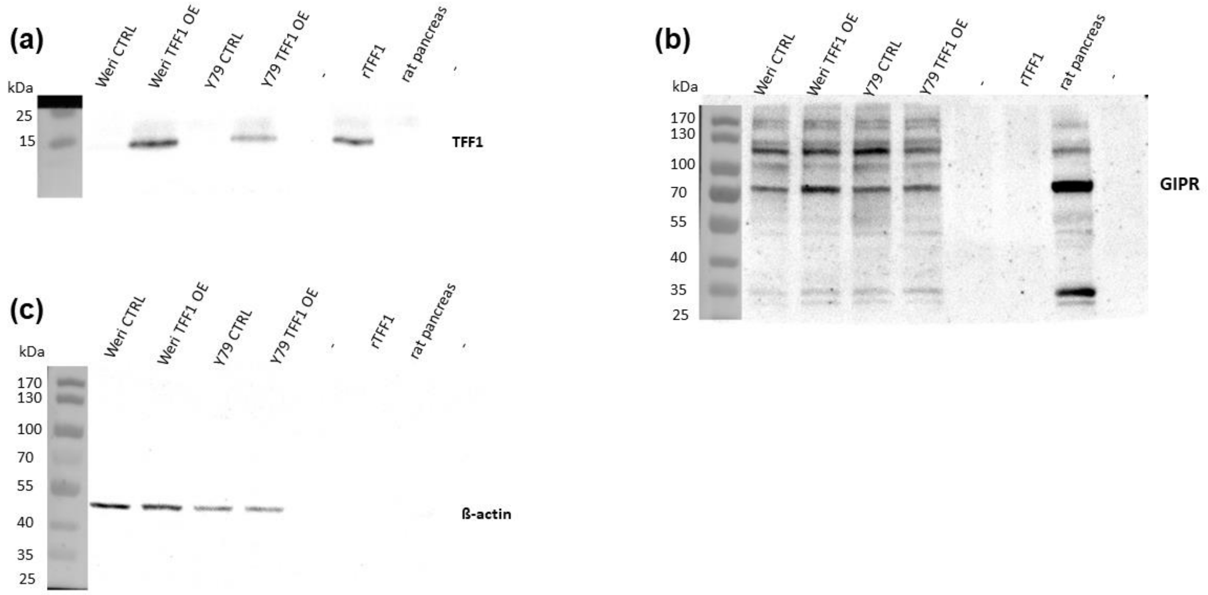


**Figure S2.** Uncropped Western blots shown in Figure 2c. Uncropped TFF1 (**a**), GIPR (**b**) and ß-actin (**c**) Western blots with molecular weight markers (left lanes in KDa) of Weri and Y79 RB cells after lentiviral TFF1 overexpression. Rat pancreas served as a positive control. CTRL= cells transduced with control vector; kDa= kilodalton; rTFF1= recombinant TFF1

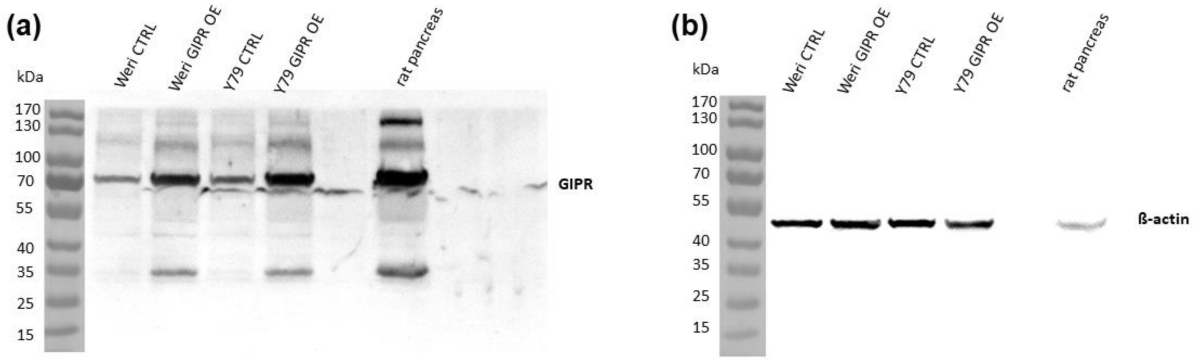


**Figure S3.** Uncropped Western blots shown in Figure 4b. Uncropped GIPR (**a**) and ß-actin (**b**) Western blots with molecular weight markers (left lanes in KDa) of Weri and Y79 RB cells after lentiviral GIPR overexpression. Rat pancreas served as a positive control. CTRL= cells transduced with control vector; kDa= kilodalton

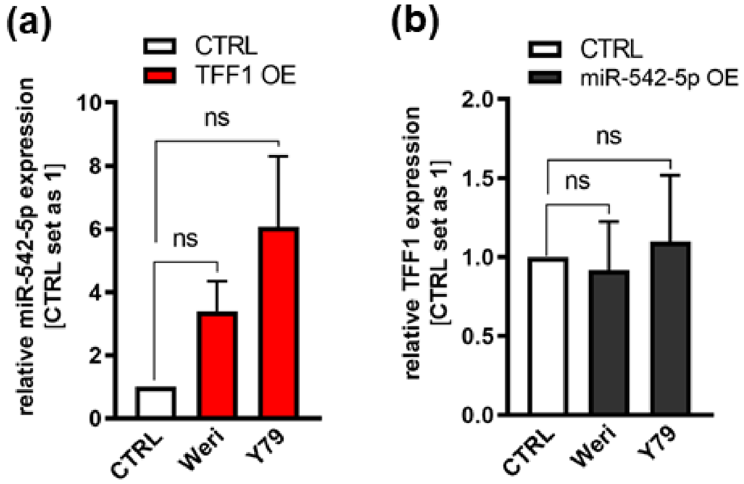


**Figure S4. Hypothetical TFF1-miR-542-5p or miR-542-5p-TFF1 signaling axis.** (**a**) Effect of TFF1 overexpression (TFF1 OE) on miR-542-5p expression levels as revealed by Real-Time PCR. **(b)** Effect of miR-542-5p overexpression (miR-542-5p OE) on TFF1 expression levels as revealed by Real-Time PCR. Values represent means ± SEM; ns= not significant compared to not overexpressing control (CTRL) cells.

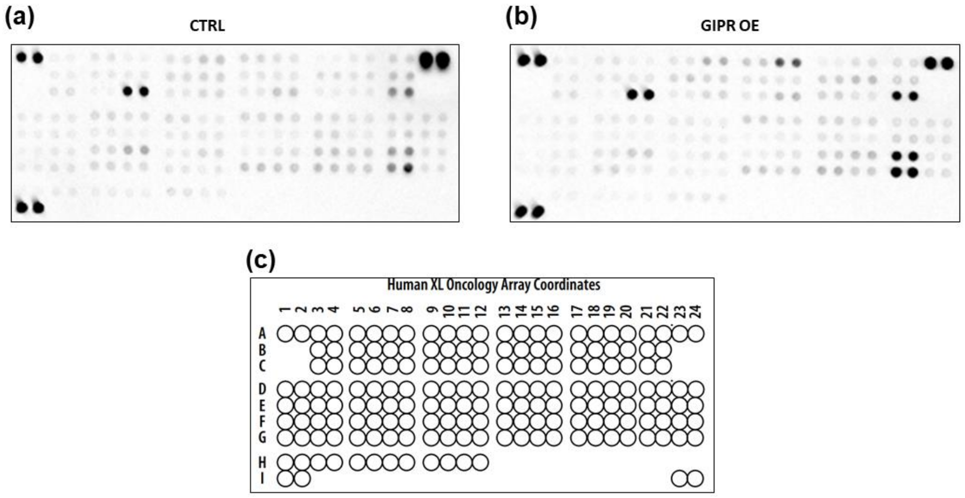


**Figure S5.** Uncropped human oncology array shown in Figure 10. Uncropped human oncology array of control cells (CTRL; **a**) and after GIPR overexpression (GIPR OE, **b**) in Weri Rb cells. The individual spots can be aligned according to a template (**c**) and the manufacturer´s data sheet at https://resources.rndsystems.com/pdfs/datasheets/ary026.pdf?v=20240424&_ga=2.25752637.1453456629.1714036456-411853202.1714036456

**Table S1.** Significantly enriched GO-terms of genes differentially expressed after GIPR overexpression in Weri RB cells. count: gene numbers enriched in a specific GO-term.

| GO-term | count | p-value |
|---|---|---|
| GO:0043066~negative regulation of apoptotic process | 5 | 0.0000084 |
| GO:0008284~positive regulation of cell proliferation | 5 | 0.0000087 |
| GO:0001934~positive regulation of protein phosphorylation | 4 | 0.00003 |
| GO:0051726~regulation of cell cycle | 4 | 0.00005 |
| GO:0042981~regulation of apoptotic process | 3 | 0.0023 |
| GO:0009410~response to xenobiotic stimulus | 3 | 0.0029 |
| GO:0008285~negative regulation of cell proliferation | 3 | 0.0079 |
| GO:0010628~positive regulation of gene expression | 3 | 0.0099 |
| GO:0045892~negative regulation of transcription | 3 | 0.0127 |
| GO:0006915~apoptotic process | 3 | 0.0139 |

**Table S2.** Significantly enriched KEGG-pathways of genes differentially expressed after GIPR overexpression in Weri RB cells. count: gene numbers enriched in a specific GO-term.

| KEGG-pathway | count | p-value |
|---|---|---|
| hsa05200:pathways in cancer | 6 | 0.0000005 |
| platinum drug resistance | 4 | 0.000011 |
| hsa04151:PI3K-Akt signaling pathway | 5 | 0.000041 |
| hsa05226:gastric cancer | 4 | 0.000096 |
| hsa05212:pancreatic cancer | 3 | 0.0012 |
| hsa05220:chronic myeloid leukemia | 3 | 0.0012 |
| hsa01521:EGFR tyrosine kinase inhibitor resistance | 3 | 0.0012 |
| hsa05222:small cell lung cancer | 3 | 0.0016 |
| hsa05215:prostate cancer | 3 | 0.0018 |
| hsa01522:endocrine resistance | 3 | 0.0018 |
| hsa04066:HIF-1 signaling pathway | 3 | 0.0022 |
| hsa04210:apoptosis | 3 | 0.0035 |
| hsa05162:measles | 3 | 0.0036 |
| hsa05224:breast cancer | 3 | 0.0041 |
| hsa05202:transcriptional misregulation in cancer 3 | 3 | 0.0070 |
| hsa05205:proteoglycans in cancer | 3 | 0.0078 |
| hsa04010:MAPK signaling pathway | 3 | 0.0165 |
| hsa05206:microRNAs in cancer | 3 | 0.0174 |
